# Impact of Social Franchising on Contraceptive Use When Complemented by Vouchers: A Quasi-Experimental Study in Rural Pakistan

**DOI:** 10.1371/journal.pone.0074260

**Published:** 2013-09-12

**Authors:** Syed Khurram Azmat, Babar Tasneem Shaikh, Waqas Hameed, Ghulam Mustafa, Wajahat Hussain, Jamshaid Asghar, Muhammad Ishaque, Aftab Ahmed, Mohsina Bilgrami

**Affiliations:** Marie Stopes Society, Karachi, Pakistan; London School of Hygiene and Tropical Medicine, United Kingdom

## Abstract

**Background:**

Pakistan has had a low contraceptive prevalence rate for the last two decades; with preference for natural birth spacing methods and condoms. Family planning services offered by the public sector have never fulfilled the demand for contraception, particularly in rural areas. In the private sector, cost is a major constraint. In 2008, Marie Stopes Society – a local NGO started a social franchise programme along with a free voucher scheme to promote uptake of IUCDs amongst the poor. This paper evaluates the effectiveness of this approach, which is designed to increase modern long term contraceptive awareness and use in rural areas of Pakistan.

**Methodology:**

We used a quasi-experimental study design with controls, selecting one intervention district and one control district from the Sindh and Punjab provinces. In each district, we chose a total of four service providers. A baseline survey was carried out among 4,992 married women of reproductive age (MWRA) in February 2009. Eighteen months after the start of intervention, an independent endline survey was conducted among 4,003 women. We used multilevel logistic regression for analysis using Stata 11.

**Results:**

Social franchising used alongside free vouchers for long term contraceptive choices significantly increased the awareness of modern contraception. Awareness increased by 5% in the intervention district. Similarly, the ever use of modern contraceptive increased by 28.5%, and the overall contraceptive prevalence rate increased by 19.6%. A significant change (11.1%) was recorded in the uptake of IUCDs, which were being promoted with vouchers.

**Conclusion:**

Family planning franchise model promotes awareness and uptake of contraceptives. Moreover, supplemented with vouchers, it may enhance the use of IUCDs, which have a significant cost attached. Our research also supports a multi-pronged approach- generating demand through counselling, overcoming financial constraints by offering vouchers, training, accreditation and branding of the service providers, and ensuring uninterrupted contraceptive supplies.

## Introduction

Family planning is one of the most effective activities in terms of costs and promoting health. It has the potential to reduce poverty and hunger, avert around 30% of maternal deaths and 10% of child deaths, thus helping to achieve the Millennium Development Goals (MDGs) [1]. Globally, government healthcare systems have faced numerous challenges in terms of governance, lack of human resources and financial constraints and frequently fail to meet the demand for family planning services. Private healthcare providers have contributed significantly to this area [2-7]. The World Health Organization (WHO) has emphasized the need to set up partnerships with private practitioners to remedying these problems, using a range of methods including social franchising [8]. Several approaches have been recommended for engaging the private sector directly in the delivery of healthcare services in low income countries, including contracting out, voucher schemes, insurance schemes, provider accreditation social marketing and social franchising [9-12].

Social franchises generally comprise of a network of the independent private health providers that use commercial franchising methods to achieve social rather than financial goals. Therefore, it is seen as a business model whereby franchising organizations provide an authorization to self-regulating providers or health service delivery outlets to operate under a specific brand name by building upon their existing expertise in the poor communities. Social franchises organize multiple, existing, private providers into contractually obligated networks. These franchisees are then provided training, branding, monitoring, and client data. They are supported to provide new or improved services, in addition to their existing patient treatment regimens through complying with a range of necessities, such as provision of certain socially beneficial services, following to quality and pricing criteria, and profit-share franchisee fees etc. A potential franchisee is typically established consequently permitting the social franchise organization to provide immediate access to its target population and facilitating a rapid scale-up. The aims of social franchising are to improve quality, increase access to care, expand the affordability of services and rapidly increase the number of delivery points for important public health services [13].

Social franchising of sexual and reproductive health services has been a successful approach for family planning programming in Asia, Africa and Latin America and has emerged as one of the possible solutions to satisfy the growing demand for quality healthcare services [14,15]. There is a great deal of interest in the developing countries around using social franchising models to promote family planning and reproductive health services in the resource poor and underserved areas [9,16]. Complementing this arrangement, the vouchers have been found to be an effective way of addressing low use of family planning services by providing an alternative way for clients to pay for providers’ fees, as well as to cover the costs of contraceptives, follow up and treatment of complications. A higher number of clients seeking family planning services can also be an added benefit to the provider’s income, as clients could consult for other ailments too.

### a) Context

In Pakistan, only 22% of the families use modern contraceptives. Among modern contraceptive, besides female sterilization, the most commonly used modern contraceptive method is the condom – a temporary, short term method with a high failure rate of (18%) with typical use [17]. The unmet need for family planning in Pakistan stands at 25% among married woman and is highest among poor women (31.1%), those living in rural areas, and women who are illiterate. As a result the country’s total fertility rate is high in the region -4.1 which is continuously increasing among the rural, illiterate and the poorest i.e. 4.5, 4.8 and 5.8 respectively [18]. Nearly 28,000 women die each year in Pakistan from causes related to the pregnancy. This number is projected to have been 1.7 times higher without contraceptive practice [19]. In order to address this high unmet need and a high fertility rate, it is essential to promote long term birth spacing methods and their use must be improved by removing the constraints and addressing the barriers associated with their uptake. Among long term contraceptive methods, the intrauterine contraceptive device (IUCD) is highly effective (>98%) and reliable in terms of averting unwanted pregnancies [20].

In Pakistan, the use of IUCD has been as low as 2%, which can be attributed to a number of factors. One major issue has been the minimal number of adequate facilities that offer IUCD services [21]. Government family planning centres are few and far between, seldom provide a conducive environment for clients and the overall state of responsiveness is poor. Supplies are only available intermittently and the providers’ attitude is not always sympathetic towards the clients’ needs. The majority of family planning centres and health facilities lack the privacy, confidentiality and hygienic conditions needed for IUCD insertion. This has turned the clients towards the private sector, where the same services are available at a higher cost, but are considered more trustworthy [22]. However, even the private providers have not been incentivized or recognized for their services and have become unmotivated. They lack inter-personal communication and counseling skills. Often they run out of stock of various contraceptives because of issues related to supply chain management. Most serious issue that prevents potential users (of public and private sector both) from adopting the IUCD as a birth spacing method is the fear of its side effects [23,24].

More than 80% of people in Pakistan have said that they would turn to a private provider for a first level healthcare (including preventive health care) and for advice on common health problems [25,26]. Moreover, franchising family planning services at the private providers’ outlets has shown positive results in terms of increasing the contraceptive rate in Pakistan [27]. However, to encourage the use of private services, there has to be a mechanism to overcome the financial barrier at the users’ end.

### b) Social franchise model – ‘Suraj’

In response to a low contraceptive prevalence and a high unmet need in the underserved rural areas of Pakistan, Marie Stopes Society (MSS) – a local non-government organization – established a social franchise model in 2008, and branded it as ‘*Suraj*’, which means ‘*sun*’ in English. The aim of the project was to provide accessible and affordable long term family planning services of a high quality by training the private providers and through marketing, branding and introducing a voucher scheme for prospective clients. So far, this network has forged partnerships with 100 female health visitors, midwives and nurses at their respective private clinics. All these providers were trained and accredited to provide condoms, emergency contraceptives, injectables, oral contraceptives and to insert and remove IUCDs. Moreover, field workers were trained to mobilize the community by paying door to door visits, by providing counselling and referrals, and by providing pre-paid IUCD vouchers to the eligible women. Women’s eligibility for vouchers was assessed using a standard tool, which had a set of nine questions including: number of meals per day; construction of house; fuel use for cooking purpose; family’s monthly income; earning member in the family; dependent family members; water source; sanitation; access to reproductive health services. These vouchers were redeemed against a free IUCD insertion, follow up visits and removal services.

## Study Methodology

### a) Study objectives

To evaluate the effectiveness of two-pronged approach using the social franchise programme and vouchers in increasing modern contraceptive awareness and its use in rural areas of Pakistan.

### b) Study area

This study was conducted in four districts across Punjab and Sindh provinces, including two intervention districts (where new social franchises were opened) and two control districts. Jhang (Punjab) and Badin (Sindh) were the intervention districts, while Khanewal (Punjab) and Dadu (Sindh) were the control districts. Study sites were selected based on the key socio-economic, demographic and reproductive health indicators. Four districts in each province were selected on the basis of poor wealth quintiles. Then one district was selected randomly for intervention in Punjab and Sindh and one for control (see Table 1).

**Table 1:** Selection of districts based on similar socio-demographic indicators, by province.

**Indicators**		**Punjab**		**Sindh**	
	**Jhang**		**Khanewal**	**Badin**	**Dadu**
**Est. district population 2011 (in thousands**)	3,599		2,626	1,463	2,175
**Annual population growth rate (1981-98**)	2.39		2.43-	2.26	2.65
**% of pop. who are female aged 15-49**	22		22	24.7	22.6
**Contraceptive Prevalence Rate**	18.1		24.2	8.0	24
**% of women who are literate**	50		56	32	42
**Average household size**	6.3		6.6	7.0	6.5
**Number of hospitals**	5		8	4	6

Source: Development Statistics Sindh & Punjab 2008; Multiple Indicator Cluster Surveys 2003 and 2007.

### c) Intervention

The intervention had a two-pronged approach – analyzing both social franchising and the voucher scheme introduced by MSS. Table 2 shows the main components of the intervention.

**Table 2:** Main intervention components.

**No**	***Suraj* social franchise components**	**Description**
1	Training on reproductive health/family planning and post training evaluation	**Medical**: reproductive health and family planning, counselling, quality of services, and IUCD insertion and removal. **Business**: basic budgeting skills, record keeping, stock management, branding, marketing and voucher management. The training is followed by post training evaluation, conducted by an external consultant (medical doctor).
2	Field worker mobilisation (FWM)	Field workers are local community members; they undergo training on family planning methods, voucher distribution systems and data recording. They pay door to door visits, raise awareness and generate referrals and distribute vouchers for IUCD to eligible women, identified through poverty scale.
3	Branding/Marketing	Providers are branded ‘Suraj’ clinics while marketing is done through FWM, posters, wall paintings, leaflets, etc. The ‘Suraj’ logo, is displayed prominently in Urdu outside all clinics.
4	Voucher for long term contraceptive method (IUCD)	Vouchers are worth PkRs 200 (US$2.27) and are only for IUCD (insertion, follow up and removal). Vouchers are distributed by field workers to eligible women. They are redeemed at a Suraj clinic; later the reimbursement is sent to the provider against her claim.

The recruitment process began with a desk review in which communities located at least 30 kilometres away from district headquarter hospitals in the study district were assessed for their needs based on multiple indicators such as total population, number of healthcare providers and demand for family planning (FP) and reproductive health (RH) services etc. After the identification of potential Private Sector Providers (PSPs), we held initial meetings with around three times more PSPs than the number actually required. Many PSPs did not meet the eligibility criteria, some were reluctant to be part of the network, a few did not attend the training, and others dropped out due to other reasons. We held an average of three face-to-face meetings with each PSP. They were informed about the Suraj network and at the same time they were assessed on following criteria:

a health facility owned or staffed by a female;
b provider lives in the same community;
c provider is interested in providing family planning services;
d provider must have formal medical qualifications;
e there must be adequate facility infrastructure (e.g. space to perform family planning services, availability of required instruments/equipment and essential amenities such as running water and electricity, and sanitation and waste disposal facilities); and
f provider must be willing to adhere to the study protocol (i.e. attend training, record keeping and reporting, and compliance with medical standards.)

The last criterion was not considered in the selection of control PSPs because they were not given any exposure such as medical training, field workers and vouchers. However, they were asked to share quarterly service statistics on a consolidated reporting format.

A total of 16 PSPs were recruited for this study and all of them were qualified Lady Health Visitors (LHVs) who is a mid-level health provider with a two year diploma in general healthcare provision and safe motherhood services. Each PSP was located approximately 30 kilometres away (in any direction) from the city center in the predominantly rural area and covered a population of 16,000-20,000. The minimum distance between any two Suraj providers was large enough to avoid the spillover effect. It is important to note that none of these providers were providing any FP services through vouchers before the baseline survey. They were new Suraj Social Franchise providers established for this study.

### d) Impact measurement

The study used a quasi-experimental pre- and post survey design with the control arm. A baseline survey was conducted in all intervention and control areas in February 2009, comprising a sample of 4,992 married women of reproductive age (MWRA, 15–49 years) living within a two to three kilometre radius of the intervention and control group service provider centre. The catchment area of each provider was mapped (allotting unique household numbers). Using a systematic approach, every second household was included, preceded by a random selection of first household. Using a ‘pick out of the hat’ method, only one MWRA was interviewed in each household. Another cross-sectional survey was conducted during July and August 2010 in the same catchment areas, 18 months after the intervention had begun. A total of 4,003 MWRAs were interviewed using the same sampling technique and strategy, with the only exception being that every fourth household was selected to maintain sample representativeness. The same sampling technique was employed in the control arm. The sample was equally divided between study arms and within each PSP’s catchment area.

### e) Data collection and management

We developed, piloted and used a structured questionnaire. The socio-demographic variables, indicators used for the evaluation of intervention included: awareness; lifetime use and current use of contraception; current use of IUCD; and unmet need for family planning. We also calculated the satisfaction of social franchise clients during the survey conducted after the intervention phase. The questionnaire was translated in Urdu and pre-tested in a different community with similar characteristics. The same questionnaire was used for the endline survey with some addition of questions pertaining to intervention. All the forms were checked for completeness, logical errors, and unclear or irrelevant responses on a daily basis. The principal and co-investigator closely monitored the entire activity to ensure data quality and adherence to the study protocol. On average, each interview took 30–35 minutes to complete. Data was double entered in Visual FoxPro version 6.0.

### f) Statistical analysis

We calculated simple frequencies and proportions for the continuous variables, which were used for the analysis of general characteristics. We used Pearson chi-square to test the association between women’s satisfaction with wealth quintile and age. For the calculation of the socio-economic index, we used principal component analysis, with variables such as: household has electricity; type of roof, wall and floor material; household water source; fuel use for cooking purpose; and the possession of household assets (television, radio, refrigerator, bicycle, car, room cooler, washing machine, motor cycle, and water pump). Since, we used a quasi-experimental design for the control arm of this study, which has a limitation of non-random assignment of individuals to control and intervention groups. Therefore, to isolate the effect of the intervention, we calculated difference-in-differences estimates.

The key reason for using difference-in-differences estimates is because the change may inherently happen over time. Therefore, having a control group allowed us to capture this inherent change (without any intervention) and to subtract it from the change brought by the intervention to find the net effect of intervention. More specifically, this method compares the change in outcome indicator in the intervention arm of the study versus the change occurring in the control arm. We calculated absolute differences in percentages from the baseline and the endline; then we worked out the net effect by subtracting the intervention absolute difference from the control. We used multi-level random intercept logistic regression to test the net effect of the intervention accounting for the observed and unobserved time-in-variant characteristics, as well as the time-varying factor between intervention and control sites.

We included communities/clusters as level two variables with multilevel modelling, which allowed us to control for intra-class correlation and cluster/community level indicators. We regressed the individual outcome against a dummy variable (created by taking the product of time [baseline and endline] by study arm [intervention and control]). We estimated a separate model to keep each contraceptive method as an outcome variable. The analysis was adjusted for other socio-demographic indicators such as wealth quintiles, education levels for women, number of members living in the house, number of living children and also the province of residence. We used Stata version 11 for descriptive analysis and models estimation.

### g) Ethics statement

Written, informed consent was taken from all the study participants. Confidentiality and anonymity was ensured. All the completed questionnaires were locked away and kept safely by the principal researcher. The study protocol including the written consent form, was reviewed and approved by the Research & Metrics Department of Marie Stopes International, London, UK and that of Marie Stopes Society Pakistan office.

## Results

A total of 4,992 married women of reproductive age (2,483 at baseline and 2,509 at endline) were interviewed in the intervention arm of the study, while 4,003 women (1,984 at baseline and 2,019 at endline) were interviewed in the control arm.

### Socio-Demographic Characteristics

Table 3 shows the characteristics of women interviewed at the baseline by intervention and control arm.

**Table 3:** Percent distribution of study participants by selected socio-demographic characteristics according to study arms at baseline.

**Characteristics**	**Intervention % (n=2,483**)	**Control % (n=2,509**)
Mean age of women in years (SD)	30.5 (5.8)	31.9 (6.6)
**No of living children**		
0-2	38.9	37.0
3-4	29.8	26.9
5+	31.3	36.2
**Women’s education level**		
No education	59.5	66.6
Primary	14.7	13.9
Middle	7.3	4.7
Secondary	10.4	7.5
Higher secondary	8.1	7.8
**Working women**		
Yes	10.5	9.7
No	89.5	90.3
**Median household members**	9	6

### a) Awareness of contraception

At the baseline, awareness regarding modern contraceptive methods was higher in the control population in comparison with intervention sites, while traditional methods (withdrawal and periodic abstinence) were more popular in the intervention sites. Pills and injections were the most commonly recognised methods in both the study arms. There was an increase in the awareness of each method within the intervention sites (see Table 4, column 5) as well as in the control sites (see Table 4, column 6). However, when compared with the control sites, the intervention population had significantly increased their awareness of any contraception method with 6.0% (p-value <0.001), modern contraception with 5.0% (p-value <0.001). The highest change was observed in male sterilisation (vasectomy) with 8.4% (p-value <0.001) and injections with 7.7% (p-value <0.001). Table 4 shows more detailed results for all types of birth spacing methods. Moreover, women cited Suraj SF providers as the source of contraceptive awareness: 69.9% had ever used contraceptives and 57.0% reported to be a current user, compared with 56.8% and 40.6% respectively of those who had heard of contraception from any other provider - p-value <0.001 for both.

**Table 4:** Awareness about contraceptive methods.

	**Intervention sites (%**)		**Control sites (%**)		**Absolute difference^1^ (% change**)		**Net effect^2^ (% change**)** (7**)
	**Baseline (1**)	**Endline (2**)	**Baseline (3**)	**Endline (4**)	**Intervention (5**)	**Control (6**)	
Any method	88.7	96.6	92.7	94.2	7.9	1.5	6.4*
Modern method	88.4	96.6	91.0	94.2	8.2	3.2	5.0*
Traditional method	69.2	84.4	58.7	64.5	15.2	5.8	9.4*
Pills	86.7	95.8	90.0	93.7	9.1	3.7	5.4*
Condom	78.7	82.6	85.1	86.6	3.9	1.5	2.4*
IUCD	84.4	94.6	87.4	93.1	10.2	5.7	4.5*
Injection	85.1	96.3	89.8	93.3	11.2	3.5	7.7*
Female sterilisation	80.9	90.1	79.4	84.9	9.2	5.5	3.7*
Male sterilisation	62.3	71.5	31.0	31.8	9.2	0.8	8.4*
Periodic abstinence	67.2	73.4	52.9	54.2	6.2	1.3	4.9*
Withdrawal	68.0	72.7	48.4	50.5	4.7	2.1	2.6*
Number of cases	2,483	1,984	2,509	2,019			

^1^ Absolute difference is the percentage changes from baseline to endline. ^2^ Net effect is the percentage change in intervention group adjusting for the percentage change in control group. Statistical significance is calculated using multiple logistic regression adjusting for socio-economic quintiles, women’s age, number of children, working women, women’s education and province. P-value: * <0.001.

### b) Ever use and current use of contraception

Table 5 shows the changes in the ever use, contraceptive prevalence rate (CPR), method mix and unmet need for contraception. At baseline, the CPR in the intervention and control sites was almost similar. At the endline survey, the CPR had increased to 48.0% (column 2) and use of modern method to 43.2%; the net effect (column 7) showed a 19.6% (p-value <0.001) increase in CPR and 22.7% (p-value <0.001) increase in modern use after adjusting by control sites. Moreover, the intervention also significantly reduced the use of traditional methods (withdrawal and periodic abstinence) by 3.1% (p-value 0.003). Among the modern methods, the highest percentage change was observed in IUCD use, with 11.4% (p-value <0.001). Column 7 also shows a statistically significant increase in the lifetime use of any contraception and of any modern method by 25.2% and 28.4% respectively. Moreover, the unmet need for contraception was reduced in both the study arms. However, when compared with the control sites, the intervention sites showed a substantial reduction in the unmet need by 7.6% (p-value <0.001).

**Table 5:** Ever use and current use of contraceptive methods.

	**Intervention sites (%**)		**Control sites (%**)		**Absolute difference^1^ (% change**)		**Net effect^2^ (% change**)** (7**)
	**Baseline (1**)	**Endline (2**)	**Baseline (3**)	**Endline (4**)	**Intervention (5**)	**Control (6**)	
**Ever use of any contraception**	**30.3**	**57.3**	**37.8**	**39.6**	**27.0**	**1.8**	25.2*
**Ever use of any modern method**	**22.1**	**53.4**	**32.8**	**35.7**	**31.3**	**2.9**	28.4*
**Contraceptive Prevalence Rate**	**27.2**	**48.0**	**28.5**	**29.7**	**20.8**	**1.2**	19.6*
**Current use**							
*Any Modern method*	*18.3*	*43.2*	*23.9*	*26.1*	*24.9*	*2.2*	22.7*
Pills	1.9	5.3	2.2	3.3	3.4	1.1	2.3*
Condom	5.4	11.4	5.8	5.3	6.0	-0.5	6.5*
Injection	2.1	6.3	4.2	5.0	4.2	0.8	3.4*
IUCD	1.9	13.7	3.0	3.4	11.8	0.4	11.4*
Female sterilization	7.0	6.5	8.9	9.2	-0.5	0.3	-0.8
Male sterilization	0.0	0.0	0.0	0.1	0.0	0.1	-0.1
*Any Traditional method*	*8.9*	*4.8*	*4.6*	*3.6*	*-4.1*	*-1.0*	-3.1*
Periodic abstinence	0.04	0.9	0.2	1.1	0.9	0.9	0.0**
Withdrawal	8.5	3.6	4.1	2.1	-4.9	-2.0	-2.9*
Others	0.4	0.4	0.3	0.3	0.0	0.0	0.0
**Unmet need for contraception**	**35.0**	**22.2**	**35.7**	**30.5**	**-12.8**	**-5.2**	-7.6*
Number of cases	2,483	1,984	2,509	2,019			

^1^ Absolute difference is the percentage change from baseline to endline^2^. Net effect is the percentage change in intervention group adjusting for the percentage change in control group. Statistical significance is calculated using multiple logistic regression adjusting for socio-economic quintiles, women’s age, number of children, working women, women’s education and province. P-value: *<0.001;**<0.01.

### c) Source of contraception

In the intervention sites, the majority of contraceptive users were receiving services from the government health facilities at baseline, then from the private health facilities, drug stores and outreach workers. At endline, more than half of the contraceptive users cited a Suraj provider as the preferred source of services. On the other hand, the share of government health facilities and outreach workers also increased from 34.1% to 45.0% and 11.7% to 19.3% respectively, as shown in Table 6.

**Table 6:** Source of contraception.

	**Intervention site**		**Control site**	
	**Baseline (n=464**)	**Endline (n=864**)	**Baseline (n=607**)	**Endline (n=535**)
Govt. health facility	44.2	15.6	34.1	45.0
Private	32.5	14.0	32.6	24.3
Outreach worker	5.8	4.5	11.7	19.3
Drugstore	9.3	8.0	11.9	4.1
Others (TBA, friend, relative)	8.2	5.7	9.7	7.3
Suraj provider	−	52.2	−	−
**Total**	**100**	**100**	**100**	**100**

### d) Share of social franchise centres and vouchers for IUCD only

A sub-group analysis of women who reported to be a current IUCD user during the endline survey showed that 76.4% of them had received insertion service from a Suraj provider, whereby 34.7% were those who received it for free (through an IUCD voucher scheme) and 41.7% paid out-of-pocket for the services. Almost a quarter of them cited other sources such as the government health facilities, private clinics or Traditional Birth Attendants (TBA).

### e) Contribution of voucher and field worker in the uptake of contraception

**Figure 1:**
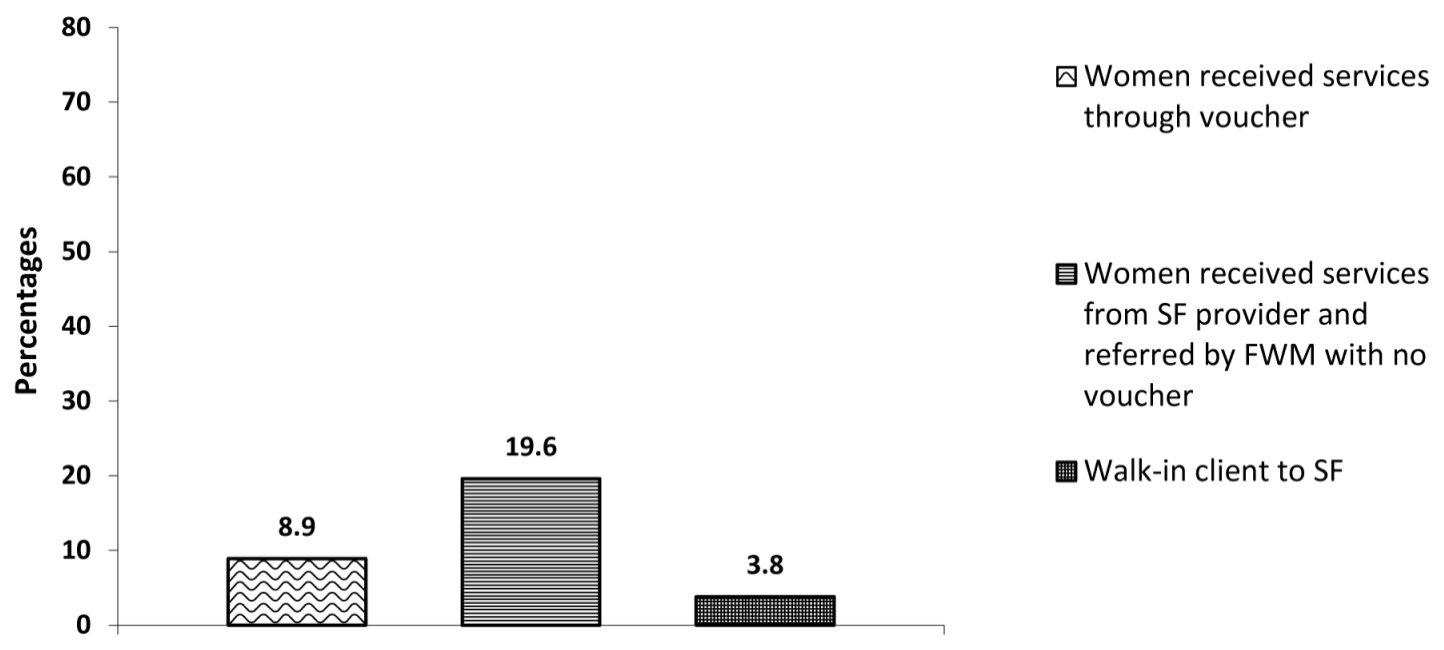
Percent distribution of women who received contraceptive services from Social franchise, by their source of motivation [Endline survey: n=1984].

Of all the women interviewed during the endline survey in the intervention sites, nearly 28% reported to have received the contraceptive services from a social franchise provider and were referred by a field worker: 8.9% were referred with vouchers (for IUCD), 20% were referred without vouchers (for any contraceptive service); while 3.8% were walk-in clients, as shown in Figure 1.

### f) Satisfaction with social franchise services

Of all the women who received services from the social franchise, 96% reported to be satisfied. The most often cited reason for being satisfied was the ‘quality of advice/information received’ (31.4%) and then the ‘affordable/cheap price’ (27.3%). Around 98% of Suraj clinics clients reported that they would recommend the services to friends and relatives. A sub-group analysis by women’s age and wealth quintile was performed, which showed that approximately 97% of women among the 15–24 age group and the 25–34 age group were satisfied, while 93.8% of women in the 35–49 age group reported satisfaction with Suraj services (p-value=0.15). There was no significant association found between wealth quintile and women’s satisfaction with Suraj services (p-value=0.69).

## Discussion

Social franchising has gained increasing attention in recent years in low income countries. However, the existing evidence has not been considered as strong enough to promote the concept [13,14,24]. To address this gap, the current study attempts to assess the effectiveness of social franchising to promote family planning, especially long term contraceptive methods, in the rural areas of Pakistan.

Although we found slight differences in the socio-economic and demographic characteristics between the intervention and control populations; the intervention had a substantive effect on the key outcome indicators: increasing the awareness and use of the modern contraceptives, more noticeably of the IUCD, and reducing the unmet need for family planning services. This is in concordance with the findings of several other studies [13-15,27]. However, given the varied socio-cultural and demographic context of different regions, it is strongly felt that the output based or performance based financing for promotion of the reproductive health services would need more research [28,29]. This domain must be explored further before making it an advocacy tool, particularly with regard to its inclusive nature and reaching out the actual poor; and also the ability of franchisees to sustain financially. Recent evidence shows that the providers need a high degree of motivation in order to serve the poor under the franchise agreement [30]. Effective monitoring, though an uphill task, is an imperative for assuring the standards, quality and responsiveness of the services delivered under the franchise model. This is the only way to build trust amongst the users of the services who then become brand ambassadors. The field workers played a key role in referring the clients to the social franchise provider in our study. Community level health workers have always been instrumental in terms of reaching out to the disadvantaged groups [31,32]. The high level of satisfaction of the women who received contraceptive services from the social franchise providers can also be considered as one of the outcomes of medical and business training and continuous monitoring of the field staff; and this has been endorsed by many other studies as well [12,13,33,34].

Despite the encouraging evidence revealed through the findings of this study, there was a certain difficulty in measuring or controlling for important confounding variables. This became even more difficult for the unmeasured confounding variables. This means that this investigation suffers some limitations such as non-random selection of intervention and control sites, as well as the effect of unmeasured confounding variables. Moreover, the study was conducted in only four districts with 16 providers, therefore generalizability may not be possible for the entire provinces.

## Conclusion

The findings of this study support the suitability of the Suraj social franchise model in promoting the awareness and use of modern contraception by increasing the accessibility to quality and affordable family planning services for underserved communities. The results reinforce the success of a two-pronged approach – generating demand through field workers and vouchers, and addressing the need through training of providers to deliver quality FP services. Our findings can be generalised in similar settings. Furthermore, it would be desirable to have an assessment of health outcomes associated with social franchise services and cost-effectiveness of this model. This would help to ascertain the effectiveness, limitations and potential of scaling up of social franchise models in other parts of the country.
