# Correction: Impact of Social Franchising on Contraceptive Use When Complemented by Vouchers: A Quasi-Experimental Study in Rural Pakistan

**DOI:** 10.1371/annotation/e230f0e7-7940-4c9a-b30e-7ceef14456d1

**Published:** 2014-01-06

**Authors:** Syed Khurram Azmat, Babar Tasneem Shaikh, Waqas Hameed, Ghulam Mustafa, Wajahat Hussain, Jamshaid Asghar, Muhammad Ishaque, Aftab Ahmed, Mohsina Bilgrami

Multiple author names were incorrectly represented in the Citation. The correct Citation is: Azmat SK, Shaikh BT, Hameed W, Mustafa G, Hussain W, et al. (2013) Impact of Social Franchising on Contraceptive Use When Complemented by Vouchers: A Quasi-Experimental Study in Rural Pakistan. PLoS ONE 8(9): e74260. doi:10.1371/journal.pone.0074260.

